# Efficacy of manual acupuncture vs. placebo acupuncture for generalized anxiety disorder (GAD) in perimenopausal women: a randomized, single-blinded controlled trial

**DOI:** 10.3389/fpsyt.2023.1240489

**Published:** 2023-10-03

**Authors:** Xin Liu, Meichen Li, Xiaoyan Xie, Yingjia Li, Keyi Li, Jingqi Fan, Jun He, Lixing Zhuang

**Affiliations:** ^1^The First Clinical Medical College, Guangzhou University of Chinese Medicine, Guangzhou, China; ^2^The Second Clinical Medical College, Guangzhou University of Chinese Medicine, Guangzhou, China; ^3^Guangdong Provincial Hospital of Chinese Medicine, Guangzhou, China; ^4^Clinical Medical College of Acupuncture Moxibustion and Rehabilitation, Guangzhou University of Chinese Medicine, Guangzhou, China; ^5^Lingnan Institute of Acupuncture and Rehabilitation, The First Affiliated Hospital of Guangzhou University of Chinese Medicine, Guangzhou, China

**Keywords:** generalized anxiety disorder, acupuncture, placebo effect, clinical trial, perimenopausal period

## Abstract

**Background:**

Generalized anxiety disorder (GAD) is common among perimenopausal women. Acupuncture may be an effective treatment for GAD, but evidence is limited. The pathogenesis of GAD is not yet clear, but it is related to the hypothalamic-pituitary-adrenal axis and its excretion, cortisol (CORT), and the adrenocorticotropic hormone (ACTH). The objective of this study is to evaluate the efficacy of manual acupuncture (MA) vs. placebo acupuncture (PA) for perimenopausal women with GAD.

**Methods:**

This study is a single-center, randomized, single-blind clinical trial conducted in the First Affiliated Hospital of Guangzhou University of Chinese Medicine. A total of 112 eligible patients with GAD were randomly assigned (1:1) to receive MA (*n* = 56) or PA (*n* = 56) three times per week for 4 weeks. The primary outcome measure was the HAMA score. The secondary outcome measures were the GAD-7 and PSQI scores and the levels of CORT and ACTH. The evaluation will be executed at the baseline, 2 weeks, the end of the treatment, and a follow-up 3-month period.

**Results:**

Significant improvements in HAMA (*p* < 0.001, *η*^2^*_p_* = 0.465), GAD-7 (*p* < 0.001, *η*^2^*_p_* = 0.359) and ACTH (*p* = 0.050) values were found between T_0_ and T_2_ in the MA group compared to the PA group. No difference in PSQI (*p* = 0.613, *η*^2^*_p_* = 0.011) and CORT (*p* = 0.903) was found between T_0_ and T_2_ in the MA group compared to the PA group. Long-term improvements in HAMA (*p* < 0.001, *p* < 0.001) were found in the MA group and PA group.

**Conclusion:**

This study was the first completed study to evaluate the efficacy of acupuncture and placebo acupuncture for GAD in perimenopausal patients. Results suggested that placebo acupuncture has a therapeutic effect, however, acupuncture had a greater therapeutic effect than placebo acupuncture. This study supports the effectiveness of acupuncture and thereby contributes to extended treatment options for GAD.

**Clinical trial registration:**http://www.chictr.org.cn, Chinese Clinical Trial Registry, ID: ChiCTR2100046604. Registered on 22 May 2021.

## 1. Introduction

Generalized anxiety disorder (GAD) is one type of mental disorder that not only includes mental symptoms like feeling on edge and irritability but also includes physical symptoms like muscle tension and sweating (1). The incidence rate of GAD during the lifetime is approximately 6.2% (2). Women are 1.5–2 times more likely to have GAD than men (3). In addition, the incidence of women aged 45–54 years old is the highest in China (4), indicating that perimenopausal women are the target population of GAD. The reason why perimenopausal women have a high risk of GAD (5) is that hormones will fluctuate violently during the perimenopausal period (6), especially the HPA axis (7). Notably, a study (8) conducted by the World Health Organization showed that less than half of patients with GAD were clinically diagnosed. However, up to 45% of patients with GAD have relevant symptoms over 2 years (9), and the diagnosis of GAD has improved. Otherwise, patients with GAD could not receive practical treatment in time. Of these, only a third of patients receive treatment. Therefore, improving the diagnosis of GAD and treating them in a timely and effective manner are methods that are urgently needed.

The first-line pharmacotherapies for GAD are serotonin reuptake inhibitors (SSRIs) and serotonin noradrenaline reuptake inhibitors (SNRIs) (10). Due to the rapid increase in serotonin after taking medicine, some patients’ conditions may be aggravated when taking SSRIs or SNRIs in the first week (11). Additionally, adverse events such as nausea, headache, and constipation (12) lead to patients refusing to take drugs. Thus, these patients seek non-drug therapies, for example, cognitive behavioral therapy (CBT). Although CBT is an effective treatment for GAD (13), the high cost (14) and lack of related professionals creates a barrier to entry for Chinese patients (15). Based on the above, alternative therapies such as acupuncture are popular among Chinese patients.

Compared with CBT (16) or SSRIs (17), acupuncture may have a therapeutic effect on anxiety, but there is no trial study assessing whether acupuncture works due to a specific effect or not. To our knowledge, no study has aimed to compare the efficacy of acupuncture vs. placebo acupuncture to clarify the specific effect of acupuncture. Hence, we designed a randomized, single-blinded controlled trial to evaluate the therapeutic efficacy of acupuncture for GAD in perimenopausal women.

## 2. Methods

### 2.1. Study design

The present study was a randomized controlled patient-and-assessor-blind trial evaluating the efficacy of acupuncture for GAD in perimenopausal women. The study was conducted from May 2021 to May 2022. All participants signed the informed consent after recruitment. The study was approved by the Ethics Committee of the First Affiliated Hospital of Guangzhou University of Chinese Medicine (K[2021]014) and had registered at the Chinese Clinical Registry (ChiCTR2100046604, http://www.chictr.org.cn). The full protocol had previously been published (18).

### 2.2. Participants

Participants were recruited from the acupuncture, gynecological, and psychological outpatient departments of the First Affiliated Hospital of Guangzhou University of Chinese Medicine. The inclusion criteria were as follows: (1) women aged between 45 and 55 years old; (2) meeting the GAD diagnosis of *Diagnostic and Statistical Manual of Mental Disorders, Fifth Edition* (DSM-5) (1); (3) Hamilton Anxiety Scale (HAMA) (19) scores ranging between 14 and 28 points; (4) no anti-anxiety drugs or other psychotropic drugs in the last 2 weeks; (5) no participation in any other research in the last month. Exclusion criteria were as follows: being (1) diagnosed with other severe illnesses such as heart failure, tumors, renal failure, and so on; (2) diagnosed with a psychotic illness such as schizophrenia, agoraphobia, and so on; (3) addicted to alcohol or drugs in the past 3 months; (4) scared of acupuncture; (5) pregnant or breastfeeding.

### 2.3. Sample size

In accordance with preliminary trials (20, 21), the HAMA scores of acupuncture and placebo acupuncture were 14.0 ± 3.2 and 19.4 ± 5.3, respectively, after treatment. We used PASS version 15.0 (NCSS, LLC. Kaysville, Utah, United States) to estimate the sample size with a power level of 90% and a two-sided significance level of 5%. Following our calculations, we found the minimum sample size required for the present study was 44 in each group. With a 20% withdrawal rate, we planned to recruit a total of 112 participants with 56 patients in each group.

### 2.4. Randomization and allocation

A random number was set as a seed by an independent researcher to generate a random allocation sequence by IBM SPSS Statistics version 26 (IBM SPSS Inc., Chicago, United States). According to the sequence, participants were randomized 1:1 into the manual acupuncture (MA) group and placebo acupuncture (PA) group.

### 2.5. Blinding

**Figure 1:**
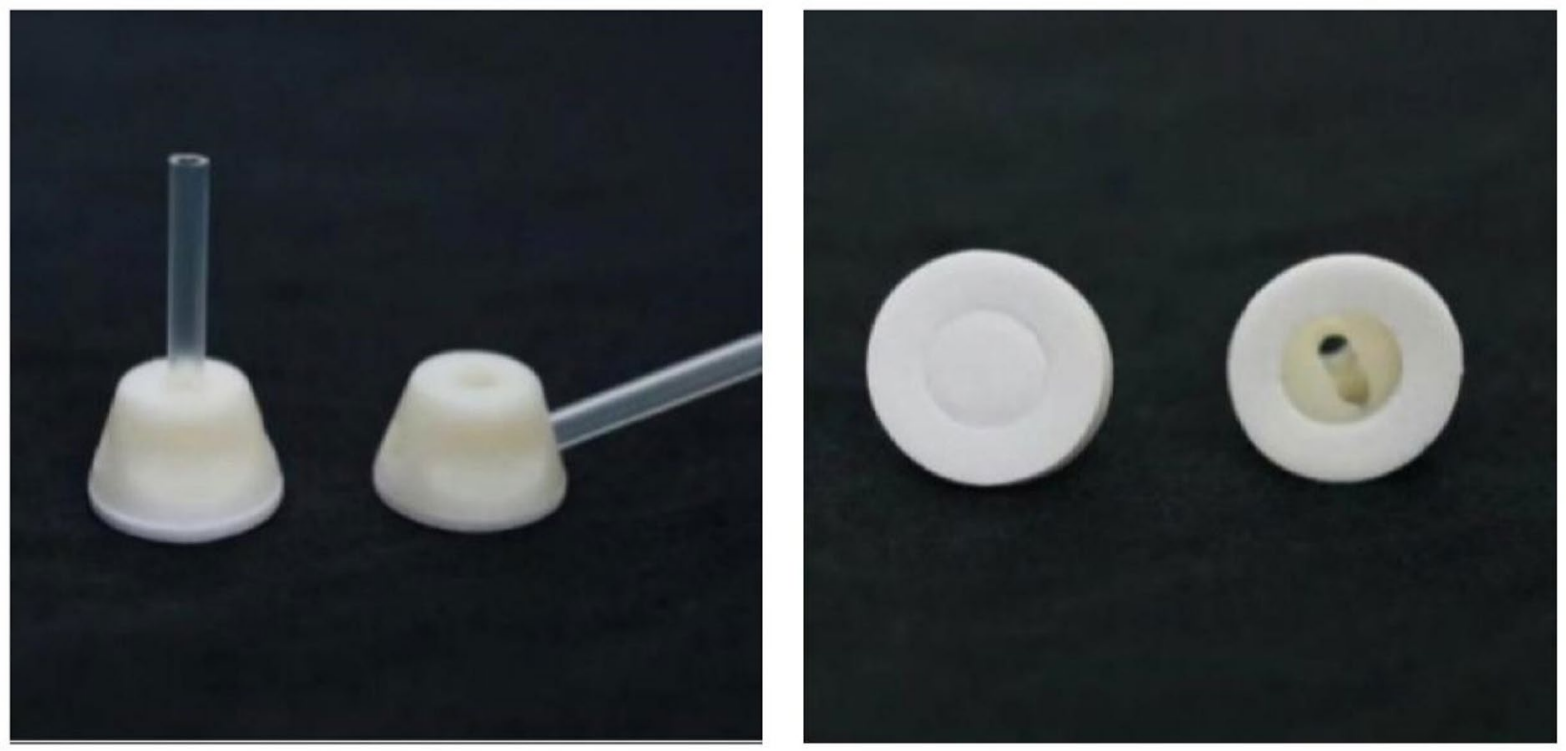
Acupuncture appliance.

A practical placebo acupuncture appliance (granted a patent by the China national intellectual property administration, No. ZL202121352221.7) designed by our team was used in the present study (Figure 1). The MA group used real needles and hollow-base appliances while the PA group used blunt needles and non-hollow-base appliances. There was adhesive tape below the base to ensure that the whole pedestal can adhere to the skin. Every participant had a patch over their eyes so that they could not visually decide whether the needle was blunt or not.

### 2.6. Intervention

Three licensed acupuncture therapists graduated from Guangzhou university of Chinese Medicine and had at least 3 years of clinical experience. All of them were trained to ensure that they truly understood the standard of the whole procedure. Another assistant followed the intervention procedures and stayed in the outpatient department to ensure consistency of acupuncture intervention among three acupuncture therapists. Enrolled participants were treated one-on-one by an acupuncture therapist from beginning to end. All participants received 4-week, 12-session acupuncture treatment, and each session took 30 min.

#### 2.6.1. Manual acupuncture group

Participants of the MA group received real acupuncture treatment with disposable stainless steel needles (φ0.30 mm × 40 mm, produced by Huanqiu Co. Ltd., China) through our device. The needles were inserted into the skin and stimulated participants to have a Deqi sensation. The acupoints included Sishenzhen [one of the main acupoints in Jin’s 3 Needle (22), Shenting (DU24), Yintang (DU29), Shenmen (HT7), and Sanyinjiao (SP6)]. All acupoints were selected bilaterally. The acupoint locations were determined according to the “2006 People’s Republic of China National Standard” (GB/T1234-2006). The depth of puncture was approximately 25–30 mm.

#### 2.6.2. Placebo acupuncture group

Participants in the PA group received placebo acupuncture treatment by blunt tipped placebo needles (φ0.30 mm × 40 mm, produced by Huanqiu Co. Ltd., China) that were not inserted into the skin and did not stimulate a Deqi sensation. Acupoints were the same as the MA group.

### 2.7. Outcomes

The HAMA (19), a 14-item scale, was used as the primary outcome measurement to assess the severity of anxiety. The Generalized Anxiety Disorder Scale (GAD-7) (23), a 7-item scale, was used as the secondary outcome measurement to assess the severity of GAD in particular. The Pittsburgh sleep quality index (PQSI) (24), a 19-item questionnaire, was used as a secondary outcome measurement to assess the quality of sleep. HAMA, GAD-7, and PQSI assessments were all conducted on the 1st, 14th, and 28th days of the trial, respectively. After the completion of 12 sessions, we followed patients via telephone or Wechat to view the HAMA, GAD-7, and PSQI scores in the 1st and 3rd month.

Blood serum was collected before and after acupuncture treatment. Enzyme-linked immunosorbent assays (ELISAs) were used to measure cortisol (CORT) and adrenocorticotropic hormone (ACTH). These related tests will be carried out by the clinical laboratory of the First Affiliated Hospital of Guangzhou University of Chinese Medicine.

After the final acupuncture session, acupuncturists asked participants which type of acupuncture they thought they received.

Adverse events (AEs) caused by acupuncture were truthfully recorded in case report forms (CRFs).

### 2.8. Statistical analysis

IBM SPSS Statistics version 26 (IBM SPSS Inc., Chicago, United States) was used for statistical analysis. All statistical tests were two-sided, and the significance level was at 0.05. If the patient dropped out, we followed the full analysis set (FAS), and the missing values were imputed using the last observation. For Baseline characteristics, the continuous values were calculated using the Mann–Whitney U test, and the categorical values were calculated using the Chi-square test. The HAMA, GAD-7, and PSQI scores were subjected to normality tests. If the scores obeyed a normal distribution, a one-way analysis of variance was performed. If not, the Kruskal-Wallis rank sum test was performed. Repeated measures analysis of covariance (25) was applied for repeated measure data. If the significance of the *F*-test of the univariate linear model was greater than 0.05, CORT and ACTH were subjected to covariance analysis. If not, the Kruskal-Wallis rank sum test was performed. The Cohen’s Kappa test was used to judge the consistency of the type of acupuncture participants received and the type they thought they received.

## 3. Results

### 3.1. Descriptive analysis

**Figure 2:**
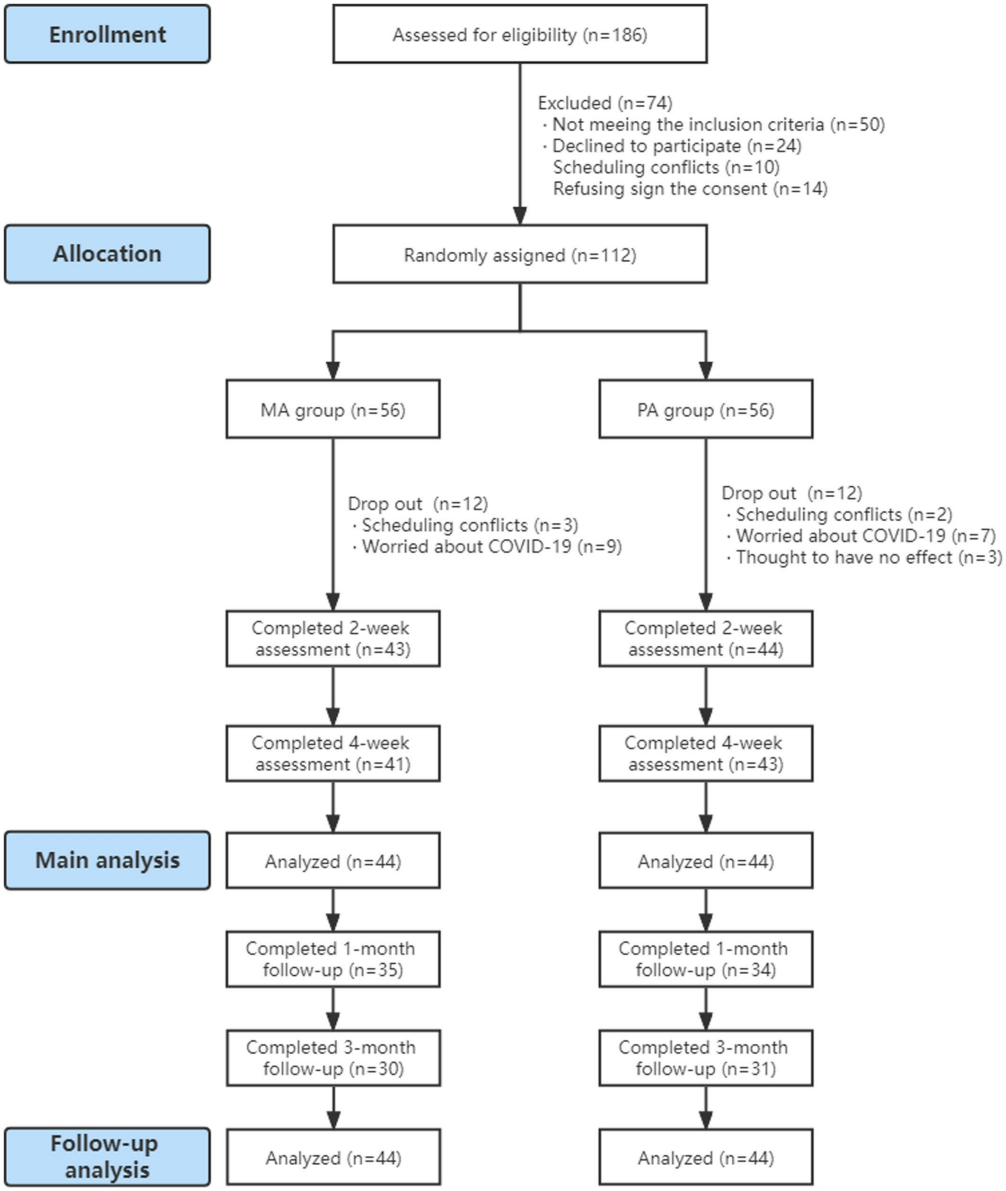
Flow chart.

We evaluated 186 potential participants for eligibility, and 112 participants were eligible and randomized (Figure 2). However, there were 12 participants who dropped out in the MA group, accounting for scheduling conflicts and worrying about COVID-19. A total of 12 participants were removed from the PA group to account for scheduling conflicts, worrying about COVID-19, and believing acupuncture has no effect. These dropped-out participants received fewer than six sessions and so our analysis excluded these participants. We analyzed 88 participants eventually. Baseline characteristics are shown in Table 1.

**Table 1:** Baseline characteristics.

Label	Levels	Groups		*p*
		MA (*n* = 44)	PA (*n* = 44)	
Age (years)	Median (IQR)	49.00 (47.00, 52.00)	50.00 (48.00, 52.00)	0.411
Course of disease (years)	Median (IQR)	3.00 (2.00, 5.00)	2.50 (1.00, 6.00)	0.351
Height (centimeter)	Median (IQR)	158.00 (155.00, 161.00)	157.00 (154.00, 160.00)	0.209
Weight (kilogram)	Median (IQR)	55.00 (50.00, 64.00)	53.50 (49.00, 56.00)	0.222
Marriage	Yes	41 (87.23%)	40 (90.90%)	0.405
	No	6 (12.77%)	4 (9.10%)	
Education	Primary school	1 (2.13%)	2 (4.55%)	0.133
	Junior high school	3 (6.38%)	3 (6.82%)	
	High school	9 (19.15%)	12 (27.27%)	
	Undergraduate	32 (68.08%)	26 (59.09%)	
	Graduate	2 (4.26%)	1 (2.27%)	
Employment status	Yes	21 (44.68%)	23 (52.27%)	0.545
	No	26 (55.32%)	21 (47.73%)	
Income	Good	3 (6.38%)	2 (4.55%)	0.603
	Middle	34 (72.34%)	33 (75.00%)	
	Bad	10 (21.28%)	9 (20.45%)	
Smoke	Yes	1 (2.13%)	2 (4.55%)	0.825
	No	46 (97.87%)	42 (95.45%)	
Alcohol	Yes	2 (4.26%)	3 (6.82%)	0.695
	No	45 (95.74%)	41 (93.18%)	

### 3.2. Outcomes analysis

#### 3.2.1. Improvements in HAMA scores in MA vs. PA

**Figure 3:**
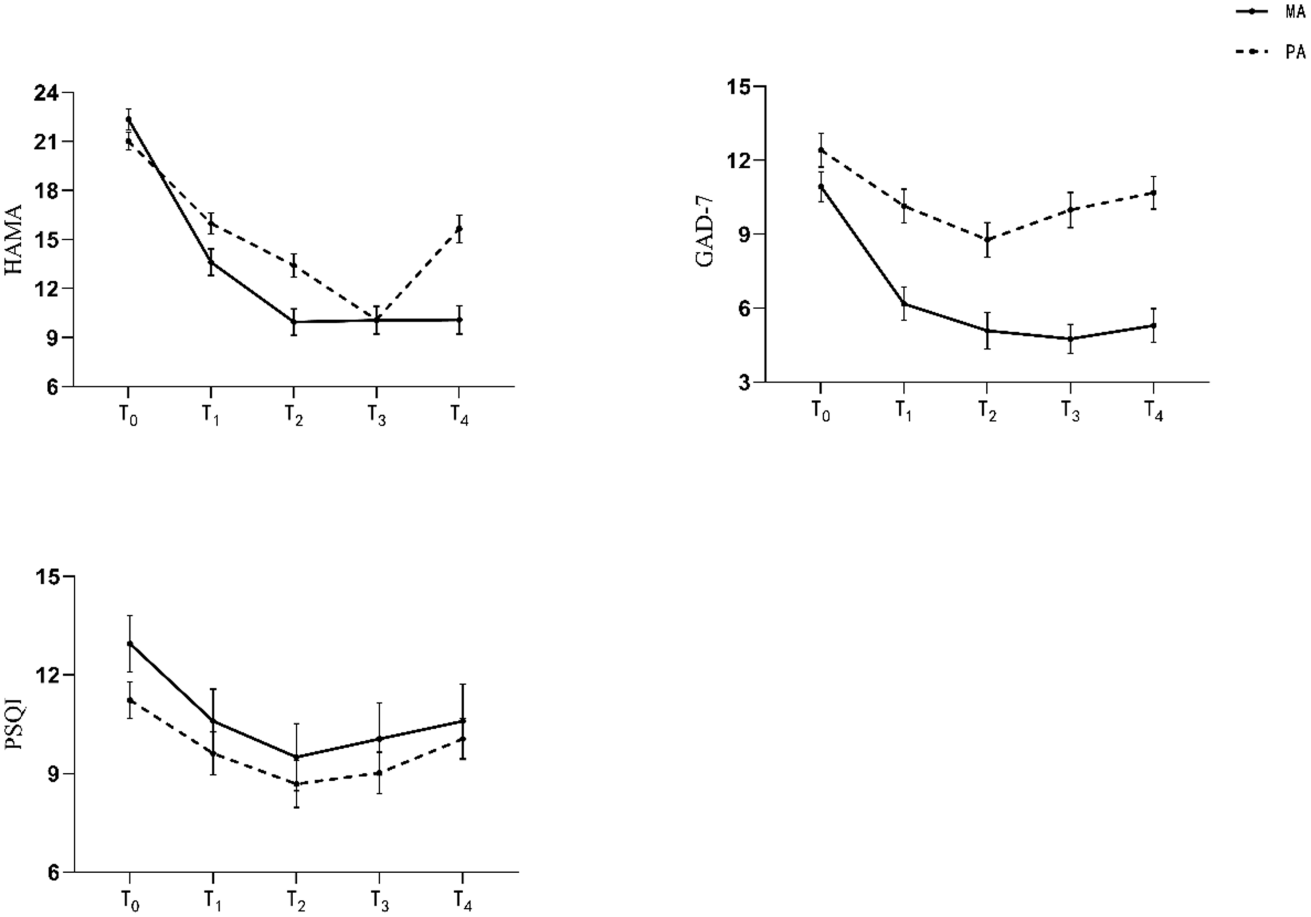
Mean scores over time.

HAMA score changes are shown in Tables 2–5 and Figure 3. Preliminary analysis revealed significant differences (*p* < 0.05) in HAMA scores from T_1_ to T_4_ between the MA and the PA group.

**Table 2:** Summary statistics for primary and secondary outcomes, and mean treatment group differences at each time of measurement.

	MA (*n* = 44)	PA (*n* = 44)	Mean difference (95% CI)	*p* value
**HAMA**				
Baseline (T_0_)	22.36 (4.25)	21.02 (3.67)	−1.34 (−3.03, 0.34)	0.117
2 weeks (T_1_)	13.61 (5.36)	15.98 (4.28)	2.36 (0.31, −4.42)	0.025
4 weeks (T_2_)	9.95 (5.36)	13.41 (4.73)	3.46 (1.31, 5.60)	0.002
1 month (T_3_)	10.05 (5.57)	10.09 (5.79)	3.57 (1.25, 5.89)	0.003
3 months (T_4_)	10.09 (5.79)	15.66 (5.62)	5.57 (3.15, 7.99)	<0.001
**GAD-7**				
Baseline (T_0_)	10.93 (3.99)	12.41 (4.57)	1.48 (−0.34, 3.30)	0.110
2 weeks (T_1_)	6.18 (4.49)	10.14 (4.57)	3.96 (2.03, 5.88)	<0.001
4 weeks (T_2_)	5.09 (4.92)	8.77 (4.67)	3.68 (1.65, 5.72)	0.001
1 month (T_3_)	4.75 (3.97)	9.98 (4.71)	5.23 (3.38, 7.07)	<0.001
3 months (T_4_)	5.30 (4.55)	10.68 (4.34)	5.39 (3.50, 7.27)	<0.001
**PSQI**				
Baseline (T_0_)	12.95 (5.71)	11.23 (3.68)	−1.73 (−3.77, 0.32)	0.096
2 weeks (T_1_)	10.59 (6.50)	9.61 (4.33)	0.98 (−3.32, 1.36)	0.409
4 weeks (T_2_)	9.50 (6.75)	8.68 (4.72)	0.82 (−3.29, 1.65)	0.512
1 month (T_3_)	10.05 (7.29)	9.02 (4.16)	1.02 (−3.54, 1.49)	0.421
3 months (T_4_)	10.59 (7.48)	10.05 (4.15)	0.55 (−3.11, 2.02)	0.673
**CORT**				
Baseline (T_0_)	12.40 (6.57)	10.45 (4.91)	1.95 (−4.41, 0.51)	0.118
4 weeks (T_2_)	9.04 (3.49)	9.15 (4.43)	0.10 (−1.59, 1.79)	0.903
**ACTH**				
Baseline (T_0_)	19.58 (10.50)	22.10 (7.22)	2.52 (−1.30, 6.34)	0.193
4 weeks (T_2_)	17.97 (8.74)	21.27 (6.70)	3.30 (0, 6.60)	0.050

**Table 3:** Effect of invention on primary and secondary outcomes.

Outcomes	Groups	Baseline (T_0_)	2 weeks (T_1_)	4 weeks (T_2_)	*p* value	*η* ^2^ *_p_*
HAMA	MA (*n* = 44)	22.36 (4.25)	13.61 (5.36)	9.95 (5.36)	<0.001	0.465
	PA (*n* = 44)	21.02 (3.67)	15.98 (4.28)	13.41 (4.73)		
GAD-7	MA (*n* = 44)	10.93 (3.99)	6.18 (4.49)	5.09 (4.92)	<0.001	0.359
	PA (*n* = 44)	12.41 (4.57)	10.14 (4.57)	8.77 (4.67)		
PSQI	MA (*n* = 44)	12.95 (5.71)	10.59 (6.50)	9.50 (6.75)	0.613	0.011
	PA (*n* = 44)	11.23 (3.68)	9.61 (4.33)	8.68 (4.72)		

*η*^2^*_p_*, partial squared eta.

**Table 4:** Follow-up results for exploratory analysis.

Outcomes	Groups	4 weeks (T_2_)	1 month (T_3_)	3 months (T_4_)	*p* value	*η* ^2^ *_p_*
HAMA	MA (*n* = 44)	9.95 (5.36)	10.05 (5.57)	10.09 (5.79)	<0.001	0.323
	PA (*n* = 44)	13.41 (4.73)	10.09 (5.79)	15.66 (5.62)		
GAD-7	MA (*n* = 44)	5.09 (4.92)	4.75 (3.97)	5.30 (4.55)	<0.001	0.324
	PA (*n* = 44)	8.77 (4.67)	9.98 (4.71)	10.68 (4.34)		
PSQI	MA (*n* = 44)	9.50 (6.75)	10.05 (7.29)	10.59 (7.48)	0.458	0.018
	PA (*n* = 44)	8.68 (4.72)	9.02 (4.16)	10.05 (4.15)		

η^2^*_p_*, partial squared eta.

**Table 5:** Long-term effect of invention on primary and secondary outcomes.

Outcomes	Groups	Baseline (T_0_)	3 months (T_4_)	*p* value
HAMA	MA (*n* = 44)	22.36 (4.25)	10.09 (5.79)	<0.001
	PA (*n* = 44)	21.02 (3.67)	15.66 (5.62)	<0.001
GAD-7	MA (*n* = 44)	10.93 (3.99)	5.30 (4.55)	<0.001
	PA (*n* = 44)	12.41 (4.57)	10.68 (4.34)	0.073
PSQI	MA (*n* = 44)	12.95 (5.71)	10.59 (7.48)	0.099
	PA (*n* = 44)	11.23 (3.68)	10.05 (4.15)	0.162

To divide the treatment effect and long-term effect, we analyzed data by way of a linear model from T_0_ to T_2_ and data from T_2_ to T_4_ separately (Tables 3 4). From T_0_ to T_2_, the analysis of HAMA scores revealed a significant interaction between time and group, *F* (2, 86) = 37.397, *p* < 0.001, *η*^2^*_p_* = 0.465. From T_2_ to T_4_, the analysis of HAMA scores revealed a significant interaction between time and group, *F* (2, 86) = 20.541, *p* < 0.001, *η*^2^*_p_* = 0.323. Besides this, we compared outcomes between T_0_ and T_4_, and results revealed significant differences (*p* < 0.05) in HAMA scores of both groups.

#### 3.2.2. Improvements in GAD-7 scores in MA vs. PA

GAD-7 score changes are shown in Tables 2–5 and Figure 3. Results revealed significant differences (*p* < 0.05) in GAD-7 scores from T_1_ to T_4_ between the MA and the PA group. From T_0_ to T_2_, the analysis of GAD-7 scores revealed a significant interaction between time and group, *F* (2, 86) = 24.104, *p* < 0.001, *η*^2^*_p_* = 0.359. From T_2_ to T_4_, the analysis of GAD-7 scores revealed a significant interaction between time and group, *F* (2, 86) = 20.619, *p* < 0.001, *η*^2^*_p_* = 0.324.

Between T_0_ and T_4_, results revealed a significant difference (*p* < 0.05) in GAD-7 scores for the MA group, whereas there were no significant difference in the GAD-7 scores of the PA group.

#### 3.2.3. Changes in PQSI scores in MA vs. PA

PSQI score changes are presented in Tables 2–5 and Figure 3. There is no significant difference in PQSI scores from T_1_ to T_4_ between the MA and the PA group, indicating there was no change in sleep quality.

#### 3.2.4. ACTH and CORT levels in MA vs. PA

Blood sample outcomes are presented in Table 2. Results revealed a significant difference (*p* = 0.05) in ACTH from T_0_ to T_2_ between the MA and the PA group. And no significant difference on CORT from T_0_ to T_2_ between the MA and the PA group.

### 3.3. Blinding analysis

A blinding analysis is presented in Table 6. A total of 17 participants believed they received PA therapy in the MA group, while 25 participants beleived they received MA therapy in the PA group. The Cohen’s Kappa coefficient of the two groups was 0.182 (95% CI: 0, 0.374). There was no significant difference to Cohen’s kappa.

**Table 6:** Blinding analysis.

	Considered to receive MA	Considered to receive PA	Cohen’s kappa (95% CI)	*p* value
MA (*n* = 44)	27 (61.36%)	17 (38.64%)	0.182 (0, 0.374)	0.088
PA (*n* = 44)	19 (43.18%)	25 (56.82%)		

### 3.4. Adverse events

The reported adverse event was bleeding. A total of 16 participants reported bleeding after acupuncture, and acupuncturists pressed cotton swabs on the acupoints so that there was no hematoma occurred. Besides, no participants were unblinded because of bleeding. Apart from bleeding, no adverse event was reported.

## 4. Discussion

Acupuncture treatment has shown greater effect than CBT (16) or SSRIs (17) in treating anxiety in existing literature. Although the effect of acupuncture was verified, placebo acupuncture is still needed (26) to prove the specific effect of acupuncture does exist. Our study is the first completed clinical study of acupuncture vs. placebo acupuncture in treating GAD. In this present study, the blinding effect is good. We compared the consistency between the type of acupuncture participants received and the type they considered. The consistency was evaluated as perfect if κ was >0.8, good if 0.6 < κ ≤ 0.8, moderate if 0.4 < κ ≤ 0.6, fair if 0.2 < κ ≤ 0.4, and poor if κ was ≤0.2 (27). The Cohen’s kappa value is 0.182, which is less than 0.2, and the consistency was thus quite poor.

Participants in the MA group revealed higher improvements in their HAMA and GAD-7 scores at 2 weeks and 4 weeks compared with the PA group. Considering that the questionnaire outcomes were quite subjective, the minimal clinically important difference (MCID) is important to assess clinical efficacy (28). To our knowledge, we did not find the MCID of HAMA. According to previous studies (29, 30), the MCID of GAD-7 is 3.3. In this study, HAMA and GAD-7 scores declined from T_0_ to T_2_. The GAD-7 score also dropped by 5.84 after acupuncture treatment. These findings indicated a reduction in the anxious level of the MA group. It is suspected that some acupuncturists often intervene in some way and, without knowing what caused the improvement, may claim credit for the therapeutic effects (31). Therefore, placebo acupuncture is urgently needed to prove the specific effect of acupuncture. Besides, a placebo control was needed to improve the evidence level of acupuncture clinical trials (32–35) and to separate specific effects from the placebo effect and determine the efficacy of acupuncture. In this present study, we found that placebo effect did exist. HAMA scores of participants in the PA group declined even after the whole acupuncture sessions were completed in 3 months. Therefore, the level of anxiety of the PA group was reduced. As our knowledge regarding the placebo effect and its mechanism has greatly improved (36), we conjectured participants in the PA group believed that they received real acupuncture treatment and convicted themselves they were being treated. This positive conviction is similar to a kind of mental treatment (37) or Hawthorne effect (38) where a person mobilizes their own positive resources to make the placebo effect work. The existence of a placebo effect in acupuncture highlights the importance of evaluating how we can improve the therapeutic effect and make as many patients better as possible.

Notably, from T_2_ to T_4_, HAMA and GAD-7 scores were raised in the follow-up analysis. This finding indicates that the anxiety level of participants could deteriorate after acupuncture treatment. Compared with the GAD-7 scores of the MA group from baseline (T_0_) and 3 months (T_4_), we found that GAD-7 scores declined by 5.63, which is more than the MCID (29, 30) of GAD-7. From T2 to T4, the GAD-7 score in the MA group increased by 0.21, which is less than MCID of GAD-7. In addition, from T0 to T4, the GAD-7 score in the PA group declined by 1.73, which is less than MCID of GAD-7. These findings indicate that although GAD-7 scores rose in the MA group from T_2_ to T_4_, the long-term effect of acupuncture is clinically significant. Besides, the changes in GAD-7 scores in the PA group could not reach the MCID, indicating that the long-term effect of placebo acupuncture is not good. Given that GAD-7 scores are rising, we conjecture that in another timepoint after 3 months, the acupuncture efficacy may diminish gradually. We suggested that acupuncture has a long-term effect, and the interval of patients receiving acupuncture treatment should be more than 3 months.

As the prevalence of sleep disturbance significantly increased among perimenopausal women (39) and the level of anxiety may be related to sleep quality (40), we added the PSQI to evaluate sleep quality, trying to find some correlation between sleep quality and GAD. PSQI scores decreased when anxiety levels decreased. However, we found that there was no difference between the two groups. The MCID of the PSQI is 4.4 (41), and the changes in the PSQI could not meet the MCID either. Besides, there was no difference between T_0_ and T_4_, indicating that the long-term effect of improving sleeping quality was poor. Since the PSQI score increased when acupuncture treatment was terminated, the sleep quality of participants may deteriorate if acupuncture treatment is terminated. However, this finding needs further study to be verified.

The association between the Hypothalamic–pituitary–adrenal (HPA) axis and depression is a known fact in the world of psychiatry (42). The interactional relationship between depression and anxiety has long been taught (43), and they are frequently comorbidities of each other (44). The mechanism behind depression and anxiety is similar; the HPA axis is involved in the occurrence of anxiety (45). ACTH and CORT are endocrine factors of the HPA axis, and we conjectured that ACTH and CORT levels would change in participants. The levels of ACTH and CORT may indicate therapeutic effect and prognosis of GAD. Therefore, we evaluated the levels of ACTH and CORT of participants. We found that ACTH and CORT level decreased, and there was a significant difference in the ACTH levels of the two groups. This tendency to decline is the same as that of the HAMA scores of participants. We conjectured that the HPA axis may be the mechanism of acupuncture. Besides, CORT is a downstream factor of the HPA axis with a lower ACTH value, meaning it is hard to find a difference between the two groups, requiring more participants.

While this is the first study to evaluate the efficacy of acupuncture and placebo acupuncture for GAD in perimenopausal patients that has been completed, this study still has several limitations. First, the sample size of this present study was small. Second, accounting for COVID-19, 16 participants were afraid of being infected so they dropped out. We did not comfort participants enough to calm them down regarding their COVID-19 concerns. Third, the follow-up period was too short, missing the key timepoint when the therapeutic effects of acupuncture disappear.

Given the inherent nature of acupuncture, two accepted methods of placebo acupuncture exist. One involves selecting the same acupoints but not inserting the needles into the skin (46), while the other entails selecting inserted points near the acupoints and inserting the needles into the skin (47). For this study, we opted for the first placebo acupuncture method because we hypothesized that the Deqi sensation is crucial for the acupuncture effect, and the Deqi sensation requires the needles to be inserted into the skin. In theory, placebo acupuncture should be physiologically inert, yet existing studies have consistently demonstrated therapeutic effects associated with placebo acupuncture (48, 49). Similarly, our own study results also indicated that placebo acupuncture does indeed have a therapeutic effect. However, acupuncture had a greater therapeutic effect than placebo acupuncture. Long-term effects of acupuncture existed and the HAMA, GAD-7, and PSQI scores diminished gradually; future studies are therefore needed to target the best therapeutic period of acupuncture to maximize therapeutic effect. The ACTH and CORT level decreased with a decrease in the anxiety level so the HPA axis may be the mechanism of acupuncture for GAD in perimenopausal women.

According to this present study, we found that acupuncture is an effective therapy for GAD in perimenopausal women and the interval of patients receiving acupuncture treatment should be more than 3 months.

## Data availability statement

The raw data supporting the conclusions of this article will be made available by the authors, without undue reservation.

## Ethics statement

The studies involving humans were approved by the Ethics Committee of the First Affiliated Hospital of Guangzhou University of Chinese Medicine. The studies were conducted in accordance with the local legislation and institutional requirements. The participants provided their written informed consent to participate in this study.

## Author contributions

LZ conceptualized and designed the study. XL drafted the manuscript. XX evaluated the HAMA, GAD-7, and PSQI scores. JF helped with the GAD data collection. ML, YL, and KL conducted the study. JH provided methodological recommendations and will manage the research. All authors have agreed on the journal to which the article has been submitted and agree to be accountable for all aspects of the work.
